# Reminiscences and Things

**Published:** 1902-07-15

**Authors:** J. S. Cassidy

**Affiliations:** Covington, Ky.


### REMINISCENCES AND THINGS.

BY J. S. CASSIDY, D.D.S., COVINGTON, KY.

Read Before the Kentucky State Dental Association.

Some twenty years ago, more or less, an epidemic of dental pulp preservation spread throughout the profession. Extraordinary efforts were made in that laudable direction, and wonderful stories were told of the uniformly successful results following certain lines of treatment. Formulæ for the purpose were presented in and out of meeting and in print, and nearly all of them were highly recommended by eminent practitioners.

Each preparation intended for capping was so perfectly constituted that when applied as a covering to the pulp that unsophisticated organ was fooled into the belief that its brief experience of exposure to the awful heat and cold of the outer world was only a horrible dream. Whether the pulp realized later on that its former sufferings were not a dream was decided—too often in the affirmative, alas—by the other dentist.

Preposterous as it seems, according to our present knowledge of physiology, the suggestion was made in good faith that we could supply nutriment to the pulp from the outside, and, for instance, lacto-phosphate of lime was for quite a while the most plausible and favorite preparation for this purpose. Once at a state meeting the writer heard a prominent member enthusiastically contend that it was good practice to cut into the dentin for the proper food, and permit the fine powder thus formed by the sharp bur to fall upon the hungry pulp like "the gentle dew from heaven upon the thirsty earth."

Then came the idea of amputating the superfluous exposed tissue, so the "flap operation" was described, also the subsequent union by first intention of the healthy excised edges.

About this period our fellow member, Dr. Wm. Van Antwerp, on leaving his office one summer afternoon for his home in the country, bought a porterhouse steak for supper. After going a little way he noticed the brown paper wrapped around it was softening through the warmth of his hands and the influence of the meat juice. He happened to be passing near a cluster of paw paw bushes, so re-enforced the weakening paper with a few leaves. Perhaps the doctor unconsciously remembered the custom immemorial among the Indians of placing for a time the beef from any tough old buffalo between fresh leaves of the paw paw to make it more tender. At any rate he was not alarmed, as others might have been, when he discovered on reaching home that his stake presented in spots a peculiar semi-digested appearance where it had become in contact with the leaves. On the contrary, the incident made him happy in the thought that perhaps the paw paw might supply us with a remedy that would supersede the existing methods of doing away with undesirable portions of the dental pulp. In a paper on "Papain" read before the International Dental Congress at Paris last year, Dr. Harlan gave Kentucky full credit for introducing the practical idea of pulp digestion, but so far as the writer knows, Dr. Harlan is entitled to the credit of first using papain for the purpose suggested.

Paipain is obtained from the common paw paw (carica papaya). The best variety probably comes from the milky juice of the unripe fruit. It is a white powder, soluble in water and glycerol; the latter preserves it from degeneration and does not interfere with its digestive powers. Papain converts fibrin into peptone, whether the solvent solution be acid or alkalin. It is non-poisonous, neither caustic nor astringent, and on account of its digestive virtues has been used in medicine as an aid to the normal functions of the stomach, and for the removal of false membranous growths, such as form in diptheria, also to remove warts, tubercles, etc. It is inactive on healthy tissue. A more or less impure ferment, papayatin or papoid, changes starch into maltose and albuminoids into peptones. Take it all and all, the power of the paw paw to supply a good digester to either the boy in the woods or the patient under the doctor's care can not be questioned.

At the present time no strenuous effort is made to retain the dental pulp or any portion of it when exposed by disease, and it is recognized that mummifying those remains which can not easily be disposed of with instruments makes the future value of the tooth an uncertain quantity. In this accepted emergency in practice Dr. Harlan has presented us with papain, the best pulp digester thus far discovered. He suggests bathing the part with one-half percent borax solution, and then applying a paste made up as follows:

| | |
|---|---|
| Papain - - - - - | gr. j. |
| Glycerol, | |
| Hydrochloric acid (1-300) | aa. gtt. j. m. |

Let this paste stay sealed in place for from two to ten days, the time required for complete solution depending upon the quantity of dead pulp to be dissolved. Being a non-irritant, the preparation causes no uneasiness. The writer judges from limited personal knowledge that Dr. Harlan is not extravagant in his claims for papain, but whether the empty tortuous canals can be successfully filled is a matter to be decided by each individual operator.

Among the few really useful remedies of recent introduction to dental materia medica the drug known as orthoform is deserving of some consideration, as it has been used long enough to fully test its virtues. Chemically, it is the methyl-ester of para-amido-meta-oxy-benzoic acid, and therefore belongs to the benzene class of organic compounds. It is a white crystalline powder of weak acid reaction, freely soluble in alcohol, sparingly and very slowly soluble in water, without odor, and produces no immediate effect on the sense of taste. At the end of two or three minutes a slightly bitter aftertaste is noticed, followed by a gradually increasing numbness of the part. It is not a poison and may be used ad libitum. It possesses some antiseptic properties, is an excellent analgesic rather than anesthetic, and is applied to relieve pain in burns and scalds, ulcers of the tongue and other parts, after extraction of teeth, in dry socket, in pyorrhea, before and after operation, etc. For these purposes it may be employed in powder form or made into a solution or an ointment.

Just a few words in conclusion. We all gratefully recognize the fact that Dr. Miller has successfully proven for all time that lactic acid formed in the mouth is the solvent in true dental caries. To this most essential factor in the disease we have regarded as important adjuncts poorly constructed enamel, low density, uncleanliness, etc. By some thinkers, however, defective enamel and density have been eliminated from the discussion, and a few go so far as to hold that the manifest filth accompanying excessive dental caries is a consequence and not a cause. They point accordingly for proof of their opinion to the apparent immunity from decay of the teeth in the mouths of innumerable adults which never receive attention from the brush or any other antiseptic treatment. The question then naturally arises, do the vital forces govern chemical changes in the mouth in a way analogous to the government of the other parts of the body, when involved in the process of destructive metabolism? One item alone as a text may suffice—nervous tissues are alkalin in reaction, but become acid through overwork and sometimes even from gentle exercise, the acidity being due to lactic acid and some uric acid. It seems therefore that ferments are not absent even in the gray matter, and are induced to become active in their several lines of work through fatigue, for instance, or other forms of so-called neuroses.

With these brief and rather crude statements in view, and in order to keep in touch with the evolution of at least a segment in the sphere of professional thought, it appears to be worth while to ask in humility—Is the inception and continuance, and their opposite phenomena, immunity from and spontaneous arrest of dental caries due more to systemic influences rather than to purely local conditions?—*Digest.*

